# Editorial: Transcriptional Regulation of Memory

**DOI:** 10.3389/fnmol.2016.00024

**Published:** 2016-04-19

**Authors:** Benedict C. Albensi, Jelena Djordjevic

**Affiliations:** Laboratory of Synaptic Plasticity and Memory Dysfunction, Division of Neurodegenerative Disorders, Department of Pharmacology and Therapeutics, University of ManitobaWinnipeg, MB, Canada

**Keywords:** molecular, memory, transcription, epigenetic, synaptic plasticity (LTP/LTD)

Memory is the capacity for retaining and reviving information, facts, events, impressions, etc., and of recalling or recognizing previous experiences (Kandel et al., 2013). The formation of human memories involves a series of complicated biochemical processes, many of which are not fully understood. Short-term, intermediate-term, and long-term memories have different biological and molecular substrates and therefore represent distinct forms of memory. A short-term memory is the retention of information for a brief time (seconds to minutes) without creation of the neural changes for later recall. In contrast, a long-term memory (hours to weeks to years) occurs when changes in neural pathways result in the storage of information that can be recalled weeks, months, or even years later. There are thousands of molecules presumably involved in memory. However, recent data implicate several families of transcription factors and epigenetic processes that appear critical in the regulation of memory. Transcription factors are already well-known to regulate the basal process of transcription, the selective activation of genes, and/or the repression of genes. Albeit, we further acknowledge the importance of transcriptional processes for both memory consolidation and reconsolidation, and recognize there are still a lot of unanswered questions: For example, what are the key transcription factors involved in the regulation of memory? What are their gene targets and how do they mediate memory formation? How is transcription regulated over time? How does epigenetics contribute to memory? Can the context or our experience influence the pattern of transcription and regulate the formation of memory?

To this end, emerging from the literature are examples of transcription factors, transcriptional processes, mechanisms of epigenetics that appear to be critically involved in synaptic plasticity and long term memory. Some of these include histone variant H3.3, nuclear factor kappa B (NF-kB) protein, nuclear factor (erythroid-derived 2)-like 2 (Nrf2) protein, histone deacetylase inhibitors, Gadd45beta expression, CREB-regulated transcriptional coactivator (CRTC1), CREB, and early growth response (Egr) factor protein. The existence of genetic interactions, epigenetic processes, and non-genetic mechanisms that transfer unique environmental context to the memory formation is of growing interest.

This *Frontiers in Molecular Neuroscience* Research Topic-Special Issue on the *Transcriptional Regulation of Memory* includes both reviews and experimental studies focused on transcription factors and epigenetic processes that regulate various forms of memory, sometimes in unique ways. Dr. Ted Abel and colleagues (McNally, et al.) developed a transgenic mouse line that expresses a tetO-regulated, hemagglutin (HA)-tagged histone H3.3, which they demonstrate has control over transgene expression. In this study, they characterize this mouse line during memory consolidation and evaluate the contribution of H3.3 to fear memory and motor learning. Drs. Barbra Kaltschmidt and Christian Kaltschmidt (Kaltschmidt and Kaltschmidt) discuss roles for NF-kB in structural plasticity and long-term memory. In this review, they also discuss differences in NF-kB activity in neurons versus glia related to NF-kB homeostasis and whether NF-kB activation is a *friend* or *foe*. Data from my laboratory is also presented by Dr. Snow (Snow et al.) that demonstrates training in the Morris water maze (MWM) is associated with the upregulation of the NF-kB p65 subunit, correlations with CREB, increases in the factor Nrf2, and elevations of actin protein. These data show that the MWM is associated with transcriptional changes in the hippocampus, including those related to redox regulation. Dr. Feng and colleagues (Jia et al.) go on to discuss epigenetic regulation and how the dysregulation of histone acetylation contributes to postoperative cognitive dysfunction. In this study, the HDAC inhibitor, suberoylanilide hydroxamic acid (SAHA) was shown to attenuate long term memory impairments in aging mice. Dr. Farah Lubin and colleagues (Jarome et al.) also present a study on epigenetic regulation in memory consolidation. In this case, they demonstrate a novel role for NF-kB in the regulation of Gadd45beta expression and DNA methylation during fear memory formation. Dr. Arturo Romano and his colleagues (de la Fuente et al.) also focus on NF-kB activity in their review and specifically address consolidation and reconsolidation memory phases. In addition, there is a discussion on the regulation of immediate-early and late genes by epigenetic mechanisms that determine enduring forms of memories. Moving on to a more classically recognized transcription factor pathway, Dr. Kelsey Martin and colleagues (Ch'ng et al.) present a study on the CREB-regulated transcriptional coactivator (CRTC1) in regulating learning and memory. Here they provide evidence for the sources of calcium required for the nuclear import of CRTC1 and show CRTC1 undergoes dynein-mediated and nuclear localization signal (NLS)-dependent transport along microtubules to reach the nucleus. Dr. Sylvia Ortega-Martínez also presents a paper on CREB that reviews various members of the CREB family. The review also highlights the role of CREB as a modulator of adult hippocampal neurogenesis (AHN) during memory consolidation. Finally, Dr. Joanna Williams and her colleagues (Nido et al.) present a study on the genomic response following long-term potentiation (LTP), an experimental paradigm associated with memory encoding. These data present the view that a new homeostatic state is created 24 h post LTP, by which the stability of neural networks regulated at different times parallel properties observed at the synapse.

From reading these papers, it becomes obvious that the formation of human memories involves a series of complicated biochemical processes many of which are not fully understood. The studies and reviews presented here implicate several families of transcription factors and epigenetic mechanisms that highlight the complexity associated with the transcriptional regulation of memory.

Finally we wish to thank the *Frontiers* family of open access journals for their excellent support in putting this series of reviews and studies together for this Research Topic.

## Author contributions

All authors listed, have made substantial, direct and intellectual contribution to the work, and approved it for publication.

## Funding

St. Boniface Hospital Research Foundation, Alzheimer Society of Manitoba, Natural Sciences and Engineering Council of Canada (NSERC), and Research Manitoba.

### Conflict of interest statement

The authors declare that the research was conducted in the absence of any commercial or financial relationships that could be construed as a potential conflict of interest.
